# Assessing the Status of and Impediments to Achieving the WHO Goal to Eliminate Viral Hepatitis B by 2030: A Scoping Review

**DOI:** 10.3390/vaccines14100846

**Published:** 2026-09-25

**Authors:** Charles Berabose, Ralf Clemens, Tatiana De Noronha, Sue Ann Costa Clemens

**Affiliations:** 1 Rwanda Biomedical Centre, Viral Hepatitis and Sexually Transmitted Infections, Kigali P.O. Box 7162, Rwanda; 2 Institute for Global Health, Department of Molecular and Developmental Medicine, University of Siena, 53100 Siena, Italy; clemens.ralf@outlook.com (R.C.); denoronha@ifgh.org (T.D.N.); sue.costa@unisi.it; 3 Bloomberg School of Public Health, Johns Hopkins University, Baltimore, MD 21205, USA

**Keywords:** hepatitis B, elimination, WHO30 targets, birth dose, triple elimination

## Abstract

**Background/Objectives**: In 2016, the World Health Organization (WHO) set targets to eliminate viral hepatitis as a public health threat by 2030, seeking a 90% reduction in new chronic infections and a 65% reduction in mortality relative to a 2015 baseline. Despite effective vaccines and potent antivirals, viral hepatitis mortality is rising. This review assessed the global and regional status of, and impediments to, the 2030 HBV elimination goal and compared hepatitis B virus (HBV) with hepatitis C virus (HCV) elimination trajectories. **Methods**: A scoping review was conducted following the JBI methodology and reported in accordance with PRISMA-ScR. Peer-reviewed literature, global and regional surveillance reports, and grey literature published between 2015 and 2025 were searched across five databases and multiple institutional repositories. Data were charted against WHO impact and service-coverage indicators and mapped across the six WHO regions. **Results**: Ten core records that met the search criteria were synthesized. Overall, HBV-specific progress is uneven across the care continuum and differs sharply between regions. Routine infant three-dose vaccination (HepB3) reached 84% coverage globally in 2022, with 190/194 (98%) Member States having introduced HepB3, driving HBsAg prevalence below 1% in children under five. However, the timely birth dose (HepB-BD) stagnated at 46% globally and just 14–18% in the African Region, where only a minority of countries offer a universal birth dose. Only 13% of the 254 million people with chronic HBV were diagnosed, and 2.6% were treated. The total viral hepatitis toll increased from 1.1 million (2019) to 1.3 million (2022), with 83% attributed to HBV. **Conclusions**: HBV elimination is technically feasible but operationally off-track, and progress is regionally inequitable. Effective vaccines make prevention feasible, but current antiviral therapy remains suppressive rather than curative; the principal impediments are the birth-dose gap (most acute in Africa), a collapsed diagnosis–treatment cascade, centralized care, and inadequate domestic financing. Realigning with the 2030 targets requires integrating HBV services into antenatal and primary care, decentralizing testing and treatment through task-sharing, sustained domestic investment, and continued development of curative therapies.

## 1. Introduction

Viral hepatitis has become one of the leading infectious causes of death worldwide. The 2024 WHO Global Hepatitis Report estimates that viral hepatitis, driven mainly by hepatitis B virus (HBV) and hepatitis C virus (HCV), now rivals tuberculosis as an infectious cause of mortality, accounting for approximately 1.3 million deaths in 2022 [1]. A substantial majority of these deaths (83%) are attributable to HBV, corresponding to roughly 3500 deaths per day, with a disproportionate burden among working-age adults and, to a lesser extent, children [1,2].

HBV frequently behaves as a silent epidemic: infection is often asymptomatic and progresses covertly over decades before manifesting as cirrhosis or hepatocellular carcinoma (HCC) [3].

In 2022, an estimated 254 million people were living with chronic hepatitis B, with a global HBsAg prevalence of about 3.2%; the burden is concentrated in the WHO African and Western Pacific Regions, where prevalence reaches 5.4% and 7.1%, respectively [4,5]. Despite the availability of effective vaccines as stand-alone or in combination for four decades, approximately 1.2 million new infections still occurred in 2022 [1]. The persistence of HBV is driven by its efficient transmission routes, particularly perinatal (mother-to-child) transmission at birth and horizontal transmission in early childhood, which are the principal drivers of the chronic disease burden. The probability of chronicity is inversely related to the age at acquisition: 80–90% of infants infected during the first year of life develop chronic infection, creating a durable reservoir of disease [4,6].

A defining challenge lies in virology. HCV is an RNA virus that has become curable: direct-acting antivirals (DAAs) achieve cure rates exceeding 95% with a short 8–12-week oral course [5,7]. HBV, by contrast, is a DNA virus that converts, once inside the hepatocyte nucleus, into a stable mini-chromosome known as covalently closed circular DNA (*cccDNA*) [8]. Current nucleos(t)ide analogs such as tenofovir and entecavir potently suppress viral replication but do not eradicate *cccDNA*, so treatment discontinuation reliably leads to viral rebound [7,9]. Consequently, managing chronic HBV in 254 million people requires long-term suppression rather than a finite cure, imposing a continuous and growing strain on health systems and complicating the case for sustained financing [9,10].

Viral hepatitis is caused by five hepatotropic viruses (A–E) whose public health significance differs markedly. Hepatitis A (HAV) and hepatitis E (HEV) are transmitted by the fecal–oral route and cause acute, self-limiting infection that does not become chronic; although HEV can cause outbreaks and severe disease in pregnancy, and HAV outbreaks still occur, their overall burden of chronic liver disease is limited relative to hepatitis B and C [11]. Hepatitis D (HDV) is a defective satellite virus that replicates only in HBV-infected individuals, using the hepatitis B surface antigen (HBsAg) as its envelope; HBV–HDV co-infection or super-infection markedly accelerates progression to cirrhosis and hepatocellular carcinoma, so HDV control is inseparable from HBV control [12]. Because chronic infection, and therefore the mortality burden, is driven overwhelmingly by HBV and HCV, this review concentrates on HBV within the broader elimination agenda.

Recognizing the scale of this threat, the World Health Assembly endorsed the Global Health Sector Strategy (GHSS) on viral hepatitis in 2016, with the goal of eliminating viral hepatitis as a public health threat by 2030 [8,13]. The strategy defines impact targets, a 90% reduction in new chronic HBV infections and a 65% reduction in HBV-related deaths relative to 2015, and service-coverage targets, including 90% coverage for the third dose of hepatitis B vaccine (HepB3), 90% coverage for the timely birth dose (HepB-BD), diagnosis of 90% of people with chronic HBV, and treatment of 80% of eligible diagnosed individuals [13,14]. The central paradox motivating this review is that mortality is rising despite mature and affordable biomedical tools, indicating a failure of policy, financing, and operational execution rather than of science [14,15].

Against this background, this scoping review addresses the question: what is the status of, and the impediments to, achieving the WHO goal of eliminating viral hepatitis B by 2030? The specific objectives were (i) to determine the global and regional current status against the WHO 2030 HBV elimination targets, analyzing incidence, mortality, and prevalence; (ii) to evaluate the effectiveness, implementation, and gaps of HBV vaccination strategies (HepB3 and birth dose) and their impact on HBV epidemiology before and after vaccine introduction; and (iii) to compare HBV and HCV elimination efforts to identify divergent challenges and transferable opportunities.

## 2. Materials and Methods

### 2.1. Study Design and Rationale

A scoping review was conducted following the Joanna Briggs Institute (JBI) methodology for evidence synthesis and reported in accordance with the Preferred Reporting Items for Systematic Reviews and Meta-Analyses extension for Scoping Reviews (PRISMA-ScR) [16,17]. This design was selected because HBV elimination spans multiple disciplines, policy, virology, pharmacology, and health-system strengthening, and because the evidence base is heterogeneous, encompassing prevalence data, economic modeling, and strategic frameworks such as the GHSS. A scoping approach is well suited to mapping the breadth of activity, synthesizing diverse evidence types, and identifying implementation gaps relevant to policymakers and program implementers. Consistent with scoping-review methodology, a formal critical appraisal or risk-of-bias assessment of individual sources was not undertaken. The objectives, eligibility criteria, and data-charting approach were defined a priori by the review team; the review protocol was registered on the Open Science Framework (OSF; registration DOI: https://doi.org/10.17605/OSF.IO/KV6GB). This scoping review was conducted and is reported in accordance with the PRISMA extension for Scoping Reviews (PRISMA-ScR) guidelines, and the completed PRISMA-ScR checklist is provided as Supplementary Material.

### 2.2. Population, Concept, Context, and Eligibility Criteria

The review was structured using the JBI Population–Concept–Context (PCC) framework. The Population comprised individuals affected by or at risk of HBV globally, including neonates at risk of mother-to-child transmission, adults with chronic infection (the legacy cohort), and marginalized groups such as migrants and people who inject drugs. The Concept was the elimination of HBV as a public health threat, incorporating the WHO 2030 targets, vaccination strategies, diagnostic pathways, and therapeutic interventions. The Context was the global landscape stratified by the six WHO Regions (African, Western Pacific, South-East Asian, Eastern Mediterranean, Region of the Americas, and European).

Eligible sources were primary research, systematic reviews, policy documents, and grey literature published in English between 1 January 2015 and 31 December 2025; the 2015 start aligned with the GHSS baseline. Studies focusing exclusively on hepatitis A, D, E, HIV, or HCV without disaggregated HBV data, basic-science studies of viral kinetics not linked to clinical or public-health outcomes, non-English publications, and material produced before 2015 were excluded.

### 2.3. Information Sources and Search Strategy

A three-pronged strategy maximized coverage of peer-reviewed and policy literature. Electronic databases searched were PubMed/MEDLINE, Embase, Web of Science, Scopus, and the Cochrane Library. Grey literature was retrieved from the WHO Institutional Repository for Information Sharing (IRIS), the WHO Global Health Observatory, the Polaris Observatory (CDA Foundation), the US Centers for Disease Control and Prevention (CDC), European Centre for Disease Prevention and Control technical reports, World Hepatitis Alliance policy documents, and Gavi Alliance documents. The search combined Boolean operators and Medical Subject Headings (MeSH); the core PubMed string was: (“Hepatitis B” OR “HBV” OR “Viral Hepatitis” OR “*Hepadnaviridae*”) AND (“Elimination” OR “Eradication” OR “Control” OR “2030 target”) AND (“Vaccination” OR “Antiviral Agents” OR “Health Policy” OR “Delivery of Health Care”). Data were charted against WHO impact indicators (incidence, mortality) and service-coverage indicators (vaccination, diagnosis, treatment). The final search across all databases and repositories was completed on 31 December 2025.

### 2.4. Selection of Sources of Evidence and Data Charting

All records retrieved from the electronic databases and grey literature sources were imported into the reference manager Zotero (version 7.0; Corporation for Digital Scholarship, Vienna, VA, USA), and duplicate records were removed. Screening was then conducted in two sequential stages: titles and abstracts were first screened against the eligibility criteria, and the full texts of the remaining potentially relevant records were subsequently assessed for inclusion. Screening and study selection were performed by the lead author (C.B.) and verified by the co-authors, with any uncertainties resolved through discussion until consensus was reached. The flow of records through identification, screening, eligibility assessment, and inclusion is summarized in the PRISMA-ScR flow diagram (Figure 1).

Data were charted using a structured charting form developed for this review and aligned with the WHO monitoring framework. For each included source, the following variables were charted: first author and year of publication; study design and geographical setting or scope; and key findings relating to HBV elimination status and its impediments, mapped to the WHO impact indicators (incidence, mortality, and prevalence) and service-coverage indicators (HepB3 and timely birth-dose vaccination, diagnosis, and treatment). Charting was performed by the lead author (C.B.) and verified by the co-authors.

In keeping with scoping-review methodology, the charted evidence was synthesized narratively rather than through statistical pooling or meta-analysis. Findings were tabulated (Table 1) and summarized descriptively against the WHO 2030 elimination targets and across the six WHO regions, allowing the breadth of the available evidence and the principal implementation gaps to be mapped.

## 3. Results and Discussion

### 3.1. Overview of the Evidence Base

A total of 411 records were retrieved from all databases. After deduplication and screening of titles, abstracts, and full texts, 126 unique records remained. Of these, eight reported research conducted on a global scale and satisfied at least two of the study objectives; two further eligible records were identified through supplementary searching, yielding ten core documents for synthesis (Figure 1).

The ten selected records illustrated the consistent contrast between strong preventive coverage and weak diagnosis, treatment, financing, and birth-dose performance (Table 1).

These comprised global strategic frameworks, surveillance reports, and systematic analyses published between 2015 and 2025, and are charted in Table 1. Charting the evidence against the WHO 2030 targets revealed a U-shaped performance pattern. Importantly, the horizontal axis of this pattern is the sequence of steps in the elimination cascade—birth-dose prevention, then the infant HepB3 series, then diagnosis, then treatment. Coverage peaks in the middle (HepB3) and falls at both ends, so that the shortfall from the 2030 targets is smallest for HepB3 and largest for the birth dose and for the diagnosis–treatment cascade, tracing a U (Figure 2).

### 3.2. Status Against the 2030 Elimination Targets

With respect to the global performance against the GHSS impact and service-coverage targets, only the control of early-childhood horizontal transmission is broadly on track; every other impact and service indicator is off-track, and mortality is moving in the wrong direction (Table 2).

The global health system has successfully operationalized routine infant immunization. By December 2020, 190 of 194 (98%) WHO Member States had introduced universal infant HBV vaccination, and HepB3 coverage reached 84% globally in 2022 [18,20]. This intervention drove HBsAg prevalence among children under five below the 2020 WHO threshold of 1% at the global level in 2017, signaling that the target for controlling horizontal childhood transmission is largely on track [23].

This success was substantially accelerated by the development of whole-cell (DTPw)- and later acellular (DTaP)-pertussis-based combination vaccines that incorporate the HBV antigen, allowing the infant series to be delivered through the established EPI schedule rather than as a stand-alone visit. The impact of this integration is well illustrated by Thailand, where incorporating HBV into EPI raised third-dose (HepB3) completion from roughly 55–60% to 85–90% [25]. Global averages conceal profound regional inequity, and the six WHO regions are at very different points on the elimination path (Figure 3). Infant HepB3 coverage in the Western Pacific (WPR, 90%), Americas (AMR/PAHO, 89%), South-East Asia (SEAR, 87%), Europe (EUR, 81%), Eastern Mediterranean (EMR, 80%), and Africa (AFR, 75%) [20].

Despite the success of HepB3, prevention of mother-to-child transmission remains a critical failure point. WHO validation guidance requires ≥90% birth-dose coverage to certify elimination, yet global HepB-BD coverage has plateaued at approximately 46%, and only 110/194 Member States provide a universal birth dose, leaving nearly half of the world’s newborns exposed to the most common route of chronic infection [18,26]. The gap is widest in the African Region, where birth-dose coverage is 14–18%, meaning infants there are roughly five times less likely to receive the dose than the global target implies.

The divergence is starkest for the birth dose. The WPR has effectively institutionalized it (81%), whereas AMR (60%), SEAR (51%), EUR (43%), and EMR (33%) lag, and AFR sits far behind at 16% [18].

Africa carries the greatest burden yet shows the weakest prevention of vertical transmission. By 2021, all 47 AFR countries delivered HepB3 as part of a pentavalent or hexavalent DTP-based combination vaccine, but only about 14 of 47 offered a universal birth dose, and just 2 had reached ≥90% timely birth-dose coverage; regional birth-dose coverage rose only from 10% to about 17% over 2016–2021, leaving roughly 33 million African newborns without a timely birth dose each year [6]. Africa also retains the highest chronic HBV prevalence and the slowest progress of any region, so the birth-dose gap is the single largest impediment to the global 2030 goal. Asia presents a split picture: the WPR is closest to elimination on the vaccination indicators, while parts of SEAR combine good HepB3 with incomplete birth-dose adoption. Europe, the Americas, and the Eastern Mediterranean occupy the middle ground, generally strong on HepB3 but held back by partial birth-dose programs and, in several countries, low diagnosis and treatment coverage.

The most alarming finding was the near-total collapse of the care cascade for the 254 million people living with chronic HBV. Diagnosis has stagnated at approximately 10–13% since 2016, constrained principally by the cost of nucleic-acid testing and reliance on centralized laboratory infrastructure. Only about 2.6% of eligible individuals receive antiviral treatment, far below the WHO 2030 target of 80%, a failure driven by a specialist-dependent, centralized model of care that is unfeasible in most low- and middle-income countries [19,24].

The diagnostic bottleneck is, however, increasingly tractable with decentralized technologies, and this deserves emphasis as a concrete route to improvement rather than a distant aspiration. Centralized quantitative nucleic-acid testing can be complemented by point-of-care HBV DNA assays, including qualitative or threshold-based tests that simply confirm viremia above the treatment-relevant level rather than quantifying it across a wide range, together with reflex HBV DNA testing on samples already found to be HBsAg-positive; the 2024 WHO guidelines explicitly endorse point-of-care HBV DNA and reflex approaches to widen access [27]. Crucially, treatment eligibility can also be established without HBV DNA at all. The AST-to-platelet ratio index (APRI), derived from two inexpensive and universally available blood tests, and point-of-care or portable transient elastography identify significant fibrosis or cirrhosis that defines treatment need, while elastography additionally triages patients for portal hypertension that predicts variceal bleeding. Such simple, decentralizable tools—rather than an advanced laboratory or an elastography suite in every district—offer a realistic pathway to case-finding, staging, and treatment initiation at the primary-care level, and to date have received insufficient attention from industry and academia relative to their potential impact on HBV elimination.

The modalities and efficacy of treatment further shape this shortfall. First-line nucleos(t)ide-analog therapy (typically tenofovir or entecavir) suppresses viral replication but does not clear the *cccDNA* reservoir, so it is suppressive rather than curative and generally lifelong; these technical limitations are examined in Section 3.4. This contrasts sharply with hepatitis C, where 8–12-week direct-acting antiviral (DAA) regimens achieve cure in over 95% of patients. The absence of a curative HBV therapy, together with the need for lifelong monitoring and adherence, makes the diagnostic and treatment gap especially consequential for the 2030 goal. Contrary to the 65% mortality reduction target, deaths are rising. Total viral-hepatitis mortality increased from 1.1 million (2019) to 1.3 million (2022); HBV accounts for about 83% of this burden, so HBV-specific deaths (on the order of 1.0–1.1 million per year) are themselves increasing. This reflects a demographic shift in which the burden has moved from acute infection, now declining because of vaccination, to chronic liver disease and hepatocellular carcinoma in aging, largely untreated cohorts [1,2].

Disaggregated by virus, of the 1.3 million viral-hepatitis deaths in 2022, approximately 83% (~1.1 million) were attributable to HBV and 17% (~0.22 million) to HCV; HBV also carries the larger chronic reservoir, with 254 million people living with HBV versus roughly 50 million living with HCV. Because HCV is curable with DAAs whereas HBV is not, the HCV burden is being reduced faster wherever treatment access exists, which throws the comparatively neglected HBV cascade into sharper relief.

### 3.3. Interpretation and Broader Context

The world has partially delivered on the preventive promise of HBV vaccination. HepB3 coverage reached 84% by 2022, driven by the availability of the pediatric combination vaccine incorporating HBV vaccine, 190 of 194 states introduced universal infant vaccination, and HBsAg prevalence in children under five fell below 1%, indicating that horizontal childhood transmission is broadly under control [14,20]. This has not translated into commensurate reductions in adult mortality. Birth-dose coverage remains around 46% (far lower (14–18%) in Africa), diagnosis has stagnated near 13%, and only about 2.6% of eligible people receive treatment, leaving the main drivers of cirrhosis and HCC largely unaddressed [18,19,26]. These findings explain the central paradox: as new pediatric infections fall, deaths rise, from an estimated 1.1 million in 2019 to about 1.3 million in 2022, of which 83% are attributable to HBV, directly contradicting the 65% mortality-reduction target [1,14]. The bottleneck is not the availability or efficacy of tools but their inadequate deployment within centralized, fragmented, and under-resourced systems.

The included records clarify the gap between the GHSS benchmarks and current performance. The decline in HBsAg prevalence in children under five to roughly 0.7% in 2022, although short of the <0.1% target, validates the core logic of EPI-based control [19]. In contrast, the broader impact indicators remain critically off-track, with approximately 1.2 million new HBV infections annually and mortality far above target [1,14,26].

Among 33 surveyed nations, 24 (73%) met the 2025 interim HBsAg ≤0.5% target in children under five and 18 (55%) met ≥90% HepB3, but only Japan reached the 2030 diagnosis target, and no country met the 2025 treatment target [22].

The persistent underperformance of the birth dose is among the most consequential operational failures in the elimination agenda. The contrast between high HepB3 coverage and poor birth-dose performance, especially in Africa, shows that the problem lies in health-system design at the maternal–newborn interface rather than in vaccine efficacy or availability [26]. Countries have institutionalized HepB3 within EPI, but the birth dose is constrained by the physical location of vaccines in cold-chain infrastructure, limited authorization of midwives and community health workers to administer it, and weak linkage between immunization and delivery or early post-natal care. Expansion is slowest where home births, private-sector deliveries, and humanitarian settings predominate and where out-of-cold-chain or controlled-temperature-chain policies remain limited. Vaccine-based prevention beyond infancy also remains under-utilized for susceptible adults and high-risk groups [14,19].

Although this review focused on HBV, several records situate it within the broader viral hepatitis framework that also encompasses HCV [14,15]. A defining contrast between the two infections is the stark asymmetry in the tools available to control them. HCV, an RNA virus, has highly effective DAAs with a >95% cure rate and a clearly defined treatment paradigm; HBV persists through *cccDNA*, and current therapies mainly achieve suppression rather than cure [5,24]. However, HBV is, in principle, easier to prevent at population scale because of its highly effective prophylactic vaccines and the concentration of chronicity risk around birth and early childhood. HCV programs have simplified diagnostics, decentralized treatment, and harnessed strong political commitment; HBV programs have not yet achieved comparable simplification and decentralization, particularly in low- and middle-income countries. This contrast reinforces the conclusion that the HBV deficit is largely operational and political. The preventive tools exist, but the systems to deploy them equitably at scale are lacking, and, as elaborated in Section 3.4, current antiviral therapy itself carries intrinsic technical and clinical limitations that compound these operational gaps [23,24].

A further asymmetry concerns prevention. Unlike HBV, HCV has no licensed prophylactic vaccine. The candidates that have reached clinical evaluation, notably a recombinant E1/E2 envelope–glycoprotein formulation, did not prevent chronic infection, and the success of curative DAAs has paradoxically complicated vaccine trials by making it unethical to withhold treatment from participants who seroconvert [28,29]. Various HCV vaccine candidates based on viral-vector, virus-like-particle (VLP), adjuvanted recombinant-protein, and mRNA platforms remain in late preclinical or early clinical development. However, the phase II failure of a chimpanzee-adenovirus (ChAd3-NSmut) prime and modified-vaccinia-Ankara (MVA-NSmut) boost regimen—which induced HCV-specific T-cell responses but did not reduce the incidence of chronic HCV infection compared with placebo in people who inject drugs—together with the success of curative DAA therapy and persisting uncertainty about the appropriate target population, has substantially reduced commercial interest in HCV vaccine development [30].

Were an effective HCV vaccine to emerge, its target population would not be universal infant cohorts, as for HBV, but people at continuing risk of exposure, principally people who inject drugs (PWID) and other high-incidence groups. This contrast underlines why HBV, with a safe and highly effective vaccine already in hand, is fundamentally a delivery problem rather than a discovery problem.

The observation that roughly 80% of profiled high-income countries are not on track to eliminate HCV by 2030 relates chiefly to the diagnosis and treatment ends of the cascade rather than to prevention. Even where curative direct-acting antivirals are available, case-finding among undiagnosed and marginalized populations and linkage to treatment remain the rate-limiting steps [23]. The same cascade logic applies to HBV, where the diagnosis–treatment gap is even wider, underscoring that decentralized testing, simplified treatment, and lessons transferable from HCV programs are crucial.

Despite numerous attempts, the development of therapeutic HBV vaccines, which would be a game changer, has failed mainly because of the development of immune tolerance and dysfunctional T cells impeding viral clearance [31]. However, novel vaccine platforms inducing strong T cell responses, such as mRNA and viral vectors paired with immunomodulators, are being tested in early-stage clinical development as a more promising concept for therapeutic vaccination [32,33]. More than three decades of therapeutic HBV vaccine development have not yet delivered a licensed product or a reproducible functional cure, and several programs have recently been discontinued. For example, Altimmune ceased development of the peptide-based candidate HepTcell in 2024 for insufficient HBsAg decline, and major manufacturers terminated multiple immunomodulatory HBV programs over 2023–2024. Encouragingly, a recent signal has emerged from combination approaches: the recombinant PreS1/S2/S protein vaccine BRII-179 restored HBV-specific B and T cell responses and, when combined with small-interfering-RNA therapy, was associated with markedly higher rates of HBsAg loss than antiviral therapy alone, reinforcing the view that therapeutic vaccination is likely to succeed only as part of a combination regimen [34]. There was a recent breakthrough in the treatment of aggressive hepatitis D with the licensure of Bulevirtide, an NTCP (sodium taurocholate co-transporting polypeptide) inhibitor which prevents HDV entry into liver cells [35]. While it does not lead to viral elimination, it stabilizes liver function and slows progression in HBV patients with HDV infection [36], which is relevant for populations mainly in Central Asia and indigenous populations in the Amazon region [37].

Recurring structural impediments help explain why HBV elimination lags despite effective technologies. The centralization of hepatitis services in tertiary hospitals and specialized clinics limits access for rural, low-income, and marginalized populations. Chronic underfunding persists even as HBV rivals tuberculosis as an infectious cause of mortality [21]. Service-delivery bottlenecks, limited vaccine availability at delivery points, weak integration with maternal and child health services, and the absence of routine HBV testing in antenatal care and HIV programs directly depress birth-dose uptake and treatment initiation. The rising burden of advanced liver disease and HCC in older, previously infected cohorts underscores the cost of delayed diagnosis. Finally, the inequitable distribution of burden across the African and Western Pacific Regions and among marginalized groups interacts with structural determinants such as poverty and weak health systems, reinforcing the ethical and social justice arguments of the WHO strategies [2]. Encouragingly, emerging triple-elimination validations in the Maldives, the first validated country in 2025, and Namibia, the first high-burden African country to reach the silver tier for HBV mother-to-child transmission control, demonstrate that integrated pathways can succeed [14].

Under the WHO’s tiered ‘path to elimination’ framework for triple elimination of mother-to-child transmission (of HIV, syphilis, and HBV), high-endemicity countries can be certified at bronze, silver, or gold milestones short of full validation. For HBV, the silver tier requires ≥90% coverage of the infant three-dose series (HepB3), ≥50% coverage of the timely hepatitis B birth dose (HepB-BD), and availability of antenatal HBsAg testing in the public sector; the gold tier raises the birth-dose threshold to ≥90% and antenatal HBsAg testing to >30%, while full validation additionally requires ≤0.1% HBsAg prevalence in children aged ≤5 years [6].

In this context, “triple elimination” refers to the integrated elimination of mother-to-child transmission of HIV, syphilis, and HBV through a single antenatal and delivery platform, rather than three vertical programs. Its promise for HBV is that HBsAg screening and the timely birth dose are delivered alongside HIV and syphilis testing at the point of antenatal care and facility birth, which is especially valuable for reaching rural and low-income populations that stand-alone hepatitis services miss.

The recent milestones reflect this. The Maldives (validated in 2025) and Namibia (the first high-burden African country to reach the silver tier for HBV mother-to-child transmission control) advanced by embedding HBV into existing HIV/PMTCT and maternal and child health pathways and by pairing that integration with sustained domestic and partner financing, rather than through any new biomedical tool.

### 3.4. Technical Limitations of Current Antiviral Therapy, Vaccine Availability and Implications for Feasibility

A recurring premise in elimination discourse is that “tools exist” and that the barriers to HBV control are principally operational. For prevention, where safe and highly effective prophylactic vaccines are available, this holds. For treatment, it requires an important qualification. First-line therapy relies on nucleos(t)ide analogs (NUCs), which suppress viral replication but neither eradicate the covalently closed circular DNA reservoir nor reliably clear integrated HBV DNA; they are therefore virustatic rather than curative. Several of their limitations are intrinsic to the biology and pharmacology of the drugs and constitute technical and clinical impediments to elimination, not merely operational ones.

First, because chronic HBV is asymptomatic for decades, lifelong daily therapy is inherently vulnerable to treatment fatigue and inconsistent adherence, particularly among younger adults. Interruption—whether patient-driven or caused by drug-supply gaps—can precipitate reactivation of viral replication, immune-mediated flares and, in patients with advanced fibrosis or cirrhosis, hepatic decompensation and acute-on-chronic liver failure. A systematic review and meta-analysis of NUC cessation estimated severe withdrawal flares or hepatic decompensation in approximately 1.2% of those who stopped, and flare-related death or liver transplantation in about 0.4%, with cirrhotic patients most vulnerable [38]. A therapy that can trigger life-threatening decompensation when taken inconsistently is difficult to deploy as the backbone of a global strategy in settings where uninterrupted supply and monitoring cannot be guaranteed. Recognizing this, the 2024 WHO guidelines pair expanded treatment access with explicit strategies to promote long-term adherence and retention in care [27]. Adherence erosion also distorts program data, because individuals counted as “on treatment” may not be virologically suppressed.

Second, viral suppression does not abolish the risk of hepatocellular carcinoma (HCC). HBV DNA integrates into the host genome from the earliest phases of infection, driving insertional mutagenesis and generating oncogenic viral–host fusion transcripts that persist irrespective of serum viral load; integrated HBV sequences are detectable in roughly 90% of HBV-related HCCs [39]. Although antiviral therapy reduces—and earlier, expanded treatment may further mitigate—the accumulation of integrations and the incidence of HCC, a clinically significant residual risk remains under long-term suppression, especially in patients with cirrhosis, long-standing infection, or extensive integration. Because HCC is the principal driver of HBV-related mortality, a therapy that suppresses replication yet cannot eliminate this residual oncogenic risk cannot, on its own, deliver the mortality reductions that the 2030 targets demand; it makes lifelong surveillance an additional, open-ended requirement.

Third, the economic vulnerabilities noted earlier are not solely external constraints but flow from the design of the therapy itself. Quasi-lifelong treatment presupposes uninterrupted procurement, decades of clinical monitoring, and sustained patient engagement—conditions seldom met in low-resource settings, where the predictable results are treatment interruptions, selective access, and silent attrition. Two complementary responses follow. In the near term, treatment can be simplified and rationally targeted. The 2024 WHO guidelines expand and simplify eligibility—allowing treatment decisions from low-cost markers such as APRI where HBV DNA testing is unavailable, and broadening antiviral prophylaxis for pregnant women to interrupt the highest-risk perinatal transmission [27]—which helps prioritize those most likely to transmit or progress and reduces dependence on centralized laboratories. In the longer term, elimination will likely require transformative therapeutic advances. Finite and potentially curative regimens, namely small interfering RNA (e.g., Xalnesiran) and antisense agents such as bepirovirsen, capsid-assembly modulators, and immunomodulatory approaches including therapeutic vaccines, together with longer-acting formulations and simplified, durable algorithms are advancing through clinical development and, if successful, would relax the adherence, monitoring, and financing demands that make lifelong suppression so fragile [40]. A realistic assessment of feasibility must therefore recognize that current antiviral tools, though valuable, are incomplete. The elimination agenda depends not only on deploying today’s tools more equitably, but also on developing better ones.

## 4. Strengths and Limitations

A strength of this review is its deliberate focus on the six WHO regions and a concise set of ten core documents and systematic analyses, which together provide a coherent and updated picture of progress. Several limitations apply. First, the synthesis relied on a small number of global and regional surveillance reports and modeling studies. Second, restriction to English may have excluded important country-specific implementation studies and grey literature.

Third, as a scoping review, the design did not include a meta-analysis or formal risk-of-bias assessment. Finally, reliance on global and regional aggregates inevitably masks within-country and subnational inequities.

## 5. Conclusions and Perspectives

The global response to hepatitis B is operating in a state of strategic disequilibrium. The “easy” phase of elimination, routine infant immunization covering 84% of the birth cohort, has largely succeeded, but the “complex” phase has not. The interventions required to stop vertical transmission (the birth dose) and to prevent mortality (diagnosis and treatment of existing infections) are paralyzed by systemic impediments. The result is a prevention–care paradox in which the next generation is protected while the current generation dies at increasing rates. The rise to 1.3 million annual deaths (83% are HBV attributable) reflects not only the intrinsic limitations of a suppressive, non-curative therapeutic toolkit but also failures of policy, financing, implementation science, and the lack of scientific, community, and policymaker championship to highlight HBV as an infectious disease second only to TB. Elimination is achievable but remains constrained by both technical and political fragility—the disease burden of today is the consequence of policy failure decades ago despite available tools, and investments today will have moderate short-term impact but huge long-term benefits. Realigning with the 2030 and beyond targets requires three structural reforms rather than optional adjustments.

First, a shift from vertical silos to triple elimination. Stand-alone hepatitis programs cannot sustain the high-coverage birth dose required to interrupt transmission. The HIV–syphilis–HBV triple-elimination framework should become the standard of care in all maternal and child health settings, with HBsAg screening embedded as a non-negotiable component of antenatal care, operationally equivalent to HIV screening.

Second, a shift from centralized to decentralized, task-shifted care. Reliance on hepatologists and PCR-based diagnostics denies care to the majority of infected people. Nurses and non-specialist physicians should be authorized to initiate antiviral therapy, supported by affordable point-of-care rapid diagnostic tests for screening and triage, including point-of-care or threshold-based HBV DNA assays, the APRI score, and portable transient elastography for staging, so that testing and treatment can be delivered without centralized laboratories or specialist referral. Delivery reforms must be matched by sustained investment in research into simpler, safer, and ultimately curative regimens that would reduce the adherence, monitoring, and financing burdens of lifelong suppression and thereby improve the feasibility of elimination.

Third, a shift from external aid to domestic investment. HBV has not attracted the donor support enjoyed by HIV/AIDS, and waiting for external aid is no longer viable. Elimination should be framed as a national health security investment with a scientifically and health-economically founded investment case demonstrating long-term return; governments should integrate HBV financing into universal health coverage budgets, earmarking funds for the birth-dose vaccine and generic antivirals. With integration, decentralization, and domestic financing, the elimination of hepatitis B as a public health threat remains within reach.

## Figures and Tables

**Figure 1:**
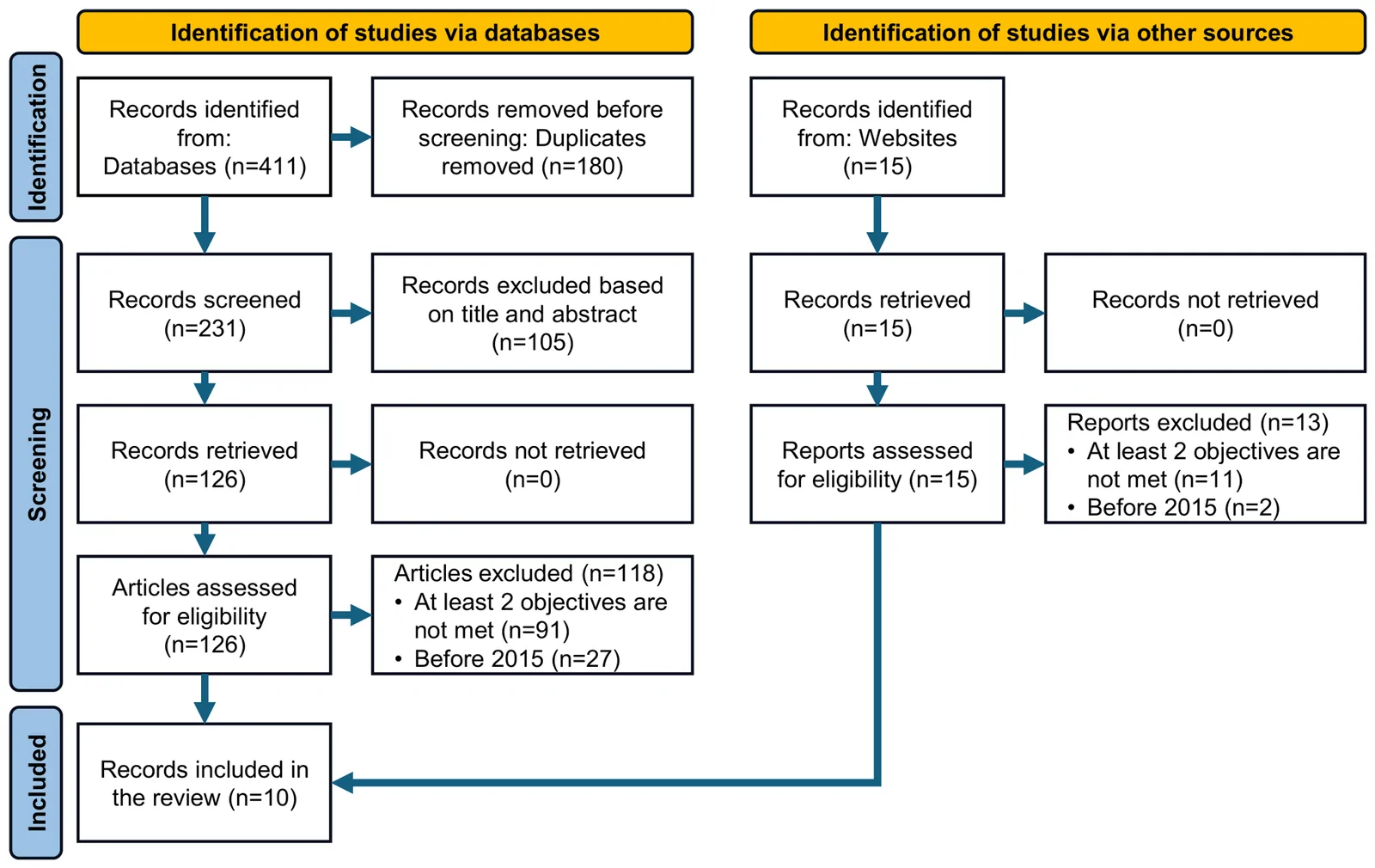
PRISMA-ScR flow diagram of the literature search, screening, and selection process.

**Figure 2:**
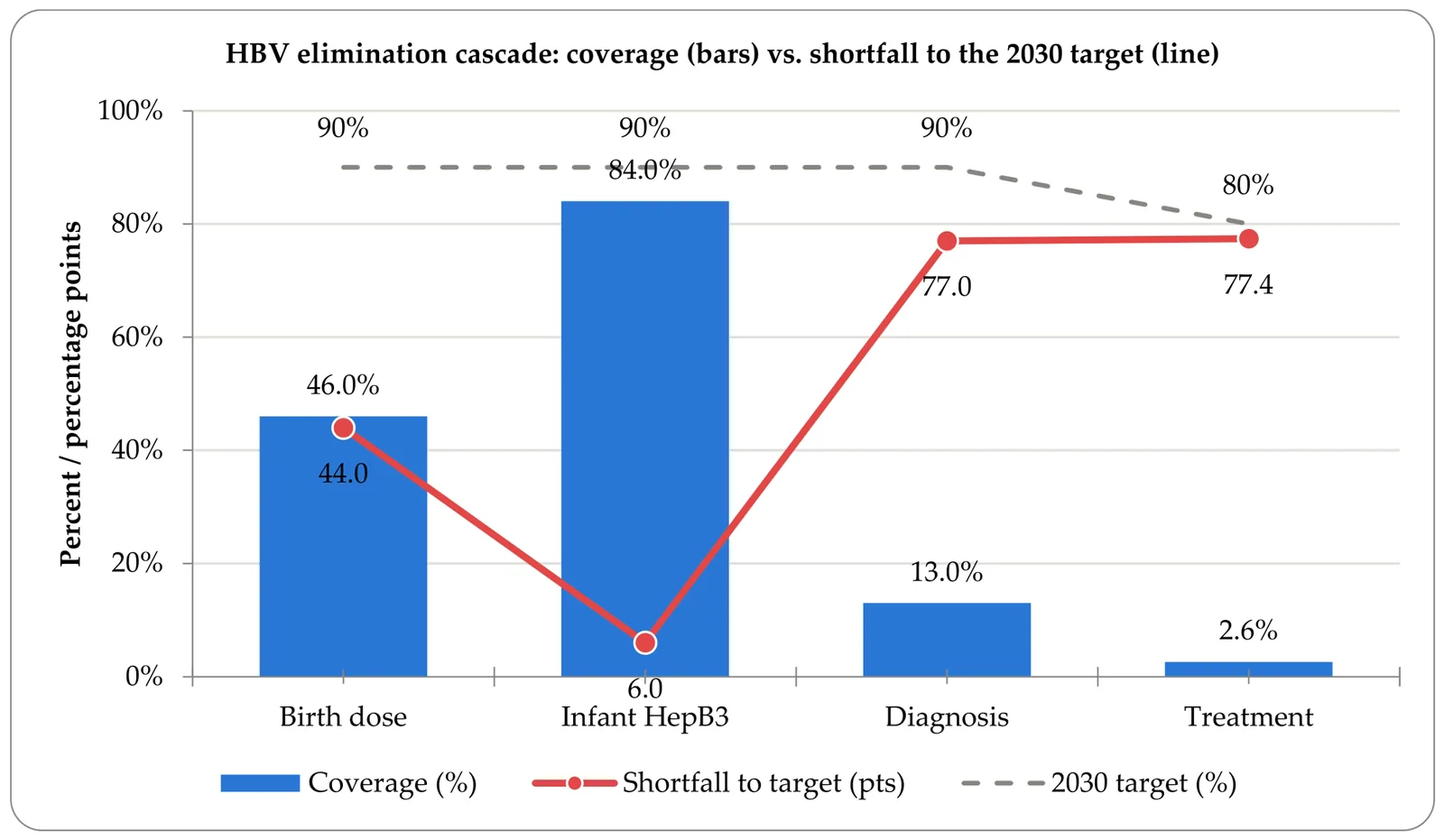
The U-shaped performance of the hepatitis B elimination cascade.

**Figure 3:**
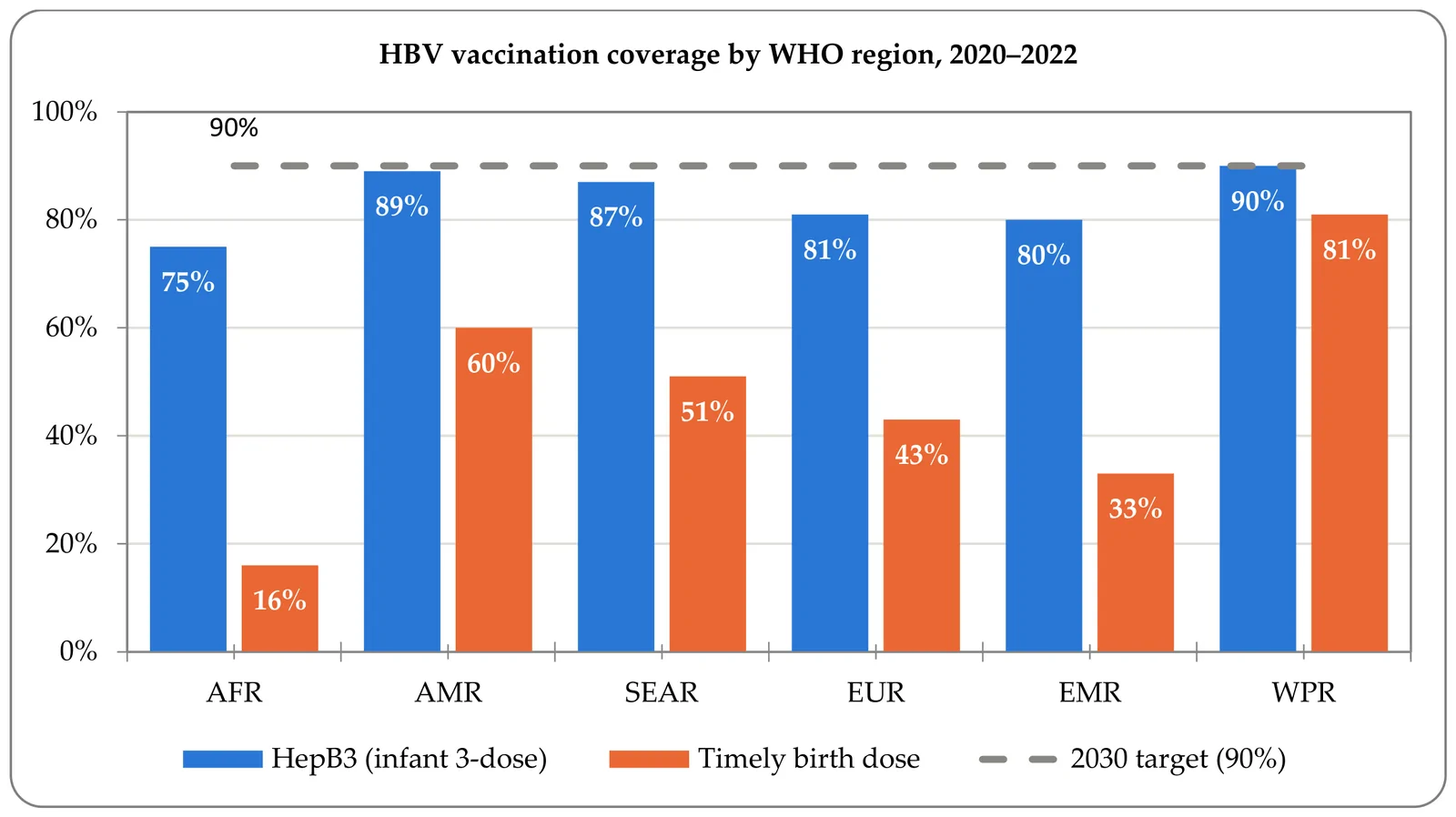
Hepatitis B vaccination coverage across the six WHO regions (2020–2022): infant three-dose series (HepB3) versus timely birth dose, against the 90% 2030 target. The African Region (AFR) shows the widest birth-dose gap. AFR, African Region; AMR, Region of the Americas; SEAR, South-East Asia Region; EUR, European Region; EMR, Eastern Mediterranean Region; WPR, Western Pacific Region. Data sources: [18,20].

**Table 1:** Synthesized data chart of the ten included records on HBV elimination status and impediments.

No.	Author, Year	Design/Setting	Key Findings (Elimination Status and Impediments)
1	Khetsuriani et al., 2022 [18]	Descriptive report; global (194 states)	In 2020, 41% of reporting countries met ≥90% coverage for both HepB-BD and HepB3; 190/194 (98%) had introduced infant HBV vaccine and 110/194 (57%) provided a universal birth dose; AFRO showed the slowest progress with the lowest coverages (Hep B3 at 73% and HepB-BD at 16%) and highest HBsAg seroprevalence (2.5%) among children aged <5 years. In the African Region, 34 (72%) of 47 countries do not provide a HepB-BD.
2	WHO, 2016 [13]	Strategic policy framework; global	82% HepB3 and 38% HepB-BD coverage by 2014; <5% of people with chronic HBV knew their status and <1% had initiated antiviral therapy.
3	WHO, 2022 [14]	Strategic policy framework; global	0.94% HBsAg prevalence among children aged 0–4 years in 2020; 10 per 100,000 HBV deaths and 20 per 100,000 new HBV infections in 2020.
4	Hsu et al., 2023 [2]	Review; global	Two-thirds of infections in WPR and AFRO; 3.8% global HBsAg prevalence (2019); 42% HepB-BD coverage (2021); 10% HBV carriers diagnosed, 2% treated (2019); deaths projected to rise 39% over 2015–2030 without action.
5	Vo-Quang & Lemoine, 2024 [19]	Review; global	~14% global HBV diagnosis and ~8% of diagnosed treated (2022) 15/194 (8%) countries achieved a 30% reduction in new infections (2015–2019); 147/194 (76%) met the 2020 HBsAg <1% target in children <5.
6	Al-Busafi & Alwassief, 2024 [20]	Review; global	HBsAg prevalence in children <5 fell from 4.7% (early 1990s) to 1.3% (2015); global HBsAg fell from >10% (1980s) to 2.9% (2020); 84% HepB3 and 46% HepB-BD coverage (2022); 3.2% global chronic HBV prevalence.
7	Howell et al., 2021 [21]	Investment framework; global	2030 targets will not be met at current investment; 194 nations endorsed the GHSS, but few developed national HBV plans or funding; 37% of states had secured domestic funding (2017), but this represents only 18% of individuals living with HBV globally; 42% of low-middle and high-income countries established domestic funding, whereas it is 25% of low-income nations; ~USD 88.7 billion needed to reach the 2030 targets.
8	Hiebert-Suwondo et al., 2025 [22]	Report; 33 countries/territories	24/33 (73%) met the 2025 interim HBsAg ≤0.5% target in children <5; 18/33 (55%) met ≥90% HepB3; only 7 (21%, where 6 of them are high-income) met the 2025 diagnosis target; Japan alone reached the 90% 2030 diagnosis target; no country met the 2025 treatment target (50% of eligible individuals).
9	Cox et al., 2020 [23]	Viewpoint; key informants	8% and 18% global HBV and HCV diagnosis rates, respectively; 80% of profiled high-income countries are not on track for HCV by 2030, with 67% requiring an additional 20 years; 84% global HepB3 coverage (2018); HBsAg <1% in children <5 reached globally in 2017.
10	Waheed, 2021 [24]	Editorial; global	Countries with national hepatitis plans rose from 17 (2012) to 124 (2019); safe blood donations rose from 89% to 97%; the annual number of clean syringes provided to PWID increased from 20 to 33 per person; HBV diagnosis improved from <5% to 10% (22% of diagnosed treated); HCV treatment scaled from 1 million to 9.4 million people.

**Table 2:** WHO 2030 HBV and HCV elimination targets versus 2022–2024 global status [1,13,19,20].

Target Area	2015 Baseline	2030 Target	HBV Status (2022–2024)	HCV Status (2022)	Status vs. Target
**Impact Targets**					
Incidence reduction	6–10 million new infections (all hepatitis)	90% reduction (<0.9 million)	1.2 million new infections	~1.0 million new HCV infections	Critically off-track
Mortality reduction	1.4 million viral hepatitis deaths (~0.9 million HBV)	65% reduction (<500,000)	1.3 million total (~1.1 million HBV)	~0.22 million HCV deaths (17% of total)	Failing (mortality increasing)
HBsAg prevalence (children <5)	1.3%	<0.1%	0.7–0.9%	N/A (HCV; no vaccine)	On track for 2025; not 2030
**Service-Coverage Targets**					
HepB3 vaccine (infants)	82–84%	90%	84%	N/A	Stagnant, off-track
HepB birth-dose vaccine	38–39%	90%	46%	N/A	Critically off-track
Diagnosis (% of CHB)	9%	90%	13.4%	36% diagnosed	Critically off-track
Treatment (% of eligible)	7%	80%	2.6%	20% treated (curative DAA)	Critically off-track

## Data Availability

No new data were created in this study. All data analyzed are available in the publicly accessible sources and databases cited in the reference list, including WHO IRIS, the WHO Global Health Observatory, and the Polaris Observatory.

## Supplementary Materials

The following supporting information can be downloaded at: https://www.mdpi.com/article/10.3390/vaccines14100846/s1, Table S1: PRISMA-ScR checklist.

## Abbreviations

The following abbreviations are used in this manuscript:

**AFRO**	WHO African Region
**AMR**	WHO Region of the Americas
**APRI**	AST-to-Platelet Ratio Index
**AST**	Aspartate Aminotransferase
**CDA**	Center for Disease Analysis
**cccDNA**	Covalently closed circular DNA
**CDC**	U.S. Centers for Disease Control and Prevention
**CHB**	Chronic hepatitis B
**DAA**	Direct-acting antiviral
**DNA**	Deoxyribonucleic acid
**DTPw/DTaP**	Whole-cell/acellular diphtheria–tetanus–pertussis combination vaccine
**EMR**	WHO Eastern Mediterranean Region
**EMTCT**	Elimination of mother-to-child transmission
**EPI**	Expanded Programme on Immunization
**EUR**	WHO European Region
**GHSS**	Global Health Sector Strategy
**HAV**	Hepatitis A virus
**HBsAg**	Hepatitis B surface antigen
**HBV**	Hepatitis B virus
**HCC**	Hepatocellular carcinoma
**HCV**	Hepatitis C virus
**HDV**	Hepatitis D virus
**HepB**	Hepatitis B vaccine
**HepB-BD**	Hepatitis B birth dose
**HepB3**	Third dose of hepatitis B vaccine
**HEV**	Hepatitis E virus
**HIV**	Human immunodeficiency virus
**IRIS**	(WHO) Institutional Repository for Information Sharing
**JBI**	Joanna Briggs Institute
**MeSH**	Medical Subject Headings
**MTCT**	Mother-to-child transmission
**NA**	Nucleos(t)ide analog
**PAHO**	Pan American Health Organization
**PCC**	Population, Concept, Context
**PMTCT**	Prevention of mother-to-child transmission
**PRISMA-ScR**	PRISMA extension for Scoping Reviews
**PWID**	People who inject drugs
**RNA**	Ribonucleic acid
**SEAR**	WHO South-East Asia Region
**TAF**	Tenofovir alafenamide
**TB**	Tuberculosis
**TDF**	Tenofovir disoproxil fumarate
**WHO**	World Health Organization
**WPR**	WHO Western Pacific Region
