# Supplementary material for: Assessing the Status of and Impediments to Achieving the WHO Goal to Eliminate Viral Hepatitis B by 2030: A Scoping Review
Source: Vaccines (Basel). 2026 Sep 25;14(10):846. doi: 10.3390/vaccines14100846 (PMC13709389; doi:10.3390/vaccines14100846)
Supplement: Supplementary file 1 [file vaccines-14-00846-s001.zip › vaccines-4549996-supplementary.pdf]

Supplementary Table S1. PRISMA-ScR Checklist

Manuscript: "Assessing the Status of and Impediments to Achieving the WHO Goal to Eliminate Viral Hepatitis B by 2030: A Scoping Review" (vaccines-4549996).

Item	PRISMA-ScR Checklist Item	Reported in (section)
**TITLE**		
1	Title — Identify the report as a scoping review.	Title ("…: A Scoping Review")
**ABSTRACT**		
2	Structured summary — Provide a structured summary (background, objectives, eligibility criteria, sources of evidence, charting methods, results, conclusions).	Abstract (Background/Objectives; Methods; Results; Conclusions)
**INTRODUCTION**		
3	Rationale — Describe the rationale for the review in the context of what is already known.	Section 1 (Introduction)
4	Objectives — Provide an explicit statement of the questions and objectives with reference to a guiding framework (e.g., PCC).	Section 1 (review question and objectives i–iii); PCC in Section 2.2
**METHODS**		
5	Protocol and registration — Indicate whether a protocol exists and if/where it can be accessed; provide registration information.	Section 2.1 (protocol defined a priori; not registered — PROSPERO does not accept scoping reviews)
6	Eligibility criteria — Specify characteristics of sources of evidence used as eligibility criteria (e.g., years, language, publication status), with rationale.	Section 2.2 (PCC; inclusion/exclusion; 2015–2025; English)
7	Information sources — Describe all information sources and the date each was last searched.	Section 2.3 (five databases; grey-literature repositories; last searched 31 Dec 2025)
8	Search — Present the full electronic search strategy for at least one database, including limits, so it could be repeated.	Section 2.3 (core PubMed/MeSH string; search completed 31 Dec 2025)
9	Selection of sources of evidence — State the process for selecting sources (i.e., screening and eligibility).	Section 2.4 (two-stage screening; reviewer and verification; Figure 1)
10	Data charting process — Describe the methods of charting data (e.g., forms; independently or in duplicate) and any process for confirming data.	Section 2.4 (structured charting form; charted by lead author, verified by co-authors)
11	Data items — List and define all variables for which data were sought and charted.	Section 2.4 (author/year; design/setting; findings mapped to WHO impact and service-coverage indicators)
12	Critical appraisal of individual sources of evidence — If done, describe methods (optional for scoping reviews).	Section 2.1 (not undertaken, consistent with scoping-review methodology)
13	Synthesis of results — Describe the methods of handling and summarizing the charted data.	Section 2.4 (narrative synthesis; tabulation; mapping to 2030 targets and six WHO regions)
**RESULTS**		
14	Selection of sources of evidence — Give numbers of sources screened, assessed for eligibility, and included, with reasons for exclusions (flow diagram).	Section 3.1 and Figure 1 (411 → 231 screened → 126 assessed → 10 included)

15	Characteristics of sources of evidence — Present characteristics of the sources charted.	Table 2 (Author/Year; Design/Setting; Key findings)
16	Critical appraisal within sources of evidence — If done, present appraisal data (optional).	Not applicable (critical appraisal not undertaken)
17	Results of individual sources of evidence — Present the charted data for each source, relevant to the review question.	Table 2; Sections 3.1–3.2
18	Synthesis of results — Summarize and/or present the charting results as they relate to the review objectives.	Sections 3.1–3.3; Tables 1–2; Figures 2–3
**DISCUSSION**		
19	Summary of evidence — Summarize the main results, linking to the review objectives, and consider their relevance to key groups.	Section 3.3 (Interpretation and Broader Context)
20	Limitations — Discuss the limitations of the scoping review process.	Section 4 (Strengths and Limitations)
21	Conclusions — Provide a general interpretation of the results with respect to objectives and potential implications.	Section 5 (Conclusions and Perspectives)
**FUNDING**		
22	Funding — Describe sources of funding and the role of funders for the review.	Funding statement (research received no external funding)

Source of checklist: Tricco, A.C.; Lillie, E.; Zarin, W.; et al. PRISMA Extension for Scoping Reviews (PRISMA-ScR): Checklist and Explanation.[16]
